# A net clinical benefit analysis of warfarin and aspirin on stroke in patients with atrial fibrillation: a nested case–control study

**DOI:** 10.1186/1471-2261-12-49

**Published:** 2012-06-26

**Authors:** Laurent Azoulay, Sophie Dell’Aniello, Teresa A Simon, David Langleben, Christel Renoux, Samy Suissa

**Affiliations:** 1 Centre for Clinical Epidemiology, Lady Davis Research Institute, Jewish General Hospital, Montreal, 3755 Côte-Sainte-Catherine, H-461, Montreal, Quebec, Canada, H3T 1E2; 2 Department of Oncology, McGill University, Montreal, Quebec, Canada; 3 Bristol-Myers Squibb, New Jersey, NJ, USA; 4 Division of Cardiology, Jewish General Hospital, Montreal, Quebec, Canada; 5 Department of Epidemiology, Biostatistics and Occupational Health, McGill University, Montreal, Quebec, Canada

**Keywords:** Atrial fibrillation, stroke, intracranial hemorrhage, warfarin, aspirin, net clinical benefit

## Abstract

**Background:**

As the management of patients treated with anticoagulants and antiplatelet drugs entails balancing coagulation levels, we evaluated the net clinical benefit of warfarin and aspirin on stroke in a large cohort of patients with atrial fibrillation (AF).

**Methods:**

A population-based cohort study of all patients at least 18 years of age with a first-ever diagnosis of chronic AF during the period 1993–2008 was conducted within the United Kingdom General Practice Research Database. A nested case–control analysis was conducted to estimate the risk of ischemic stroke and intracranial hemorrhage associated with the use of warfarin and aspirin. Cases were matched up to 10 controls on age, sex, and date of cohort entry. The adjusted net clinical benefit of warfarin and aspirin (expressed as the number of strokes prevented per 100 persons per year) was calculated by subtracting the ischemic stroke rate (prevented by therapy) from the intracranial hemorrhage (ICH) rate (increased by therapy).

**Results:**

The cohort included 70,766 patients newly-diagnosed with chronic AF, of whom 5519 experienced an ischemic stroke and 689 an ICH during follow-up. The adjusted net clinical benefit of warfarin was 0.59 (95% CI: 0.45, 0.73). However, the benefit was not seen for patients below (0.08, 95%: -0.38, 0.54) and above (−0.49, 95% CI: -1.13, 0.15) therapeutic range. The net clinical benefit of warfarin, apparent after 3 months of continuous use, increased as a function of CHADS_2_ score. The net clinical benefit was not significant with aspirin (−0.07, 95% CI: -0.22, 0.08), though it was seen in certain subgroups.

**Conclusions:**

Warfarin provides a net clinical benefit in patients with atrial fibrillation, which is maintained with longer duration of use, particularly when used within therapeutic range. A similar net effect is not as clear with aspirin.

## Background

For the past 50 years, warfarin has been the mainstay treatment for patients with atrial fibrillation (AF), a population at an increased risk of thromboembolic events [1]. Its efficacy has been well established in randomized controlled trials (RCTs), decreasing the risk of ischemic stroke by over 60% [2,3]. However, this therapy remains largely underused in clinical practice [4-6], in part because of its narrow therapeutic range, and its association with a number of important complications.

The use of this therapy must be balanced between its benefits and risks, with intracranial hemorrhage (ICH) representing the most feared complication of this therapy, an event associated with a high mortality rate [7]. Recently, two studies quantified the net clinical benefit of warfarin using ‘real world’ cohorts of patients with AF [8,9]. In the first study, Singer et al. [8] showed the net clinical benefit of warfarin was greatest among high risk patients. Their analyses included time spent outside therapeutic range (35% of warfarin exposed time), therefore questions remain as to the actual net clinical benefit for patients below, within, and above therapeutic range. Furthermore, the net clinical benefit of aspirin was not evaluated, a pharmacotherapy often used in this population. The second study by Olesen et al. [9] found a positive net clinical benefit with warfarin among patients at high risk of stroke and bleeding, and no net clinical benefit with aspirin. The authors did not have access to INR information, and thus it was not possible to assess the net clinical benefit of warfarin according to different levels of anticoagulation.

As novel oral anticoagulants are entering the market, it is imperative to identify patients most likely to benefit from warfarin and aspirin therapy. Thus, the objective of this study was to evaluate the net clinical benefit of warfarin, overall and stratified according to different levels of anticoagulation, as well as that of aspirin in a large population-based cohort of patients with chronic AF.

## Methods

### Data source

This study was conducted using the General Practice Research Database (GPRD), a primary care database from the United Kingdom (UK) [10]. It contains the complete primary care medical record for more than 10 million people enrolled in more than 600 general practices. The geographic distribution of the practices participating in the GPRD has been shown to be representative of the UK population, and age and sex distributions of patients in the GPRD are similar to those reported by the National Population Census [11]. The recorded information on diagnoses and drug exposures has been validated and proven to be of high quality [12-15]. The study protocol was approved by the Independent Scientific Advisory Committee of the GPRD and the Research Ethics Committee of the Jewish General Hospital, Montreal, Canada.

### Study population

We identified all patients, at least 18 years of age, diagnosed for the first time with chronic AF or atrial flutter between January 1, 1993 and December 31, 2008 within the GPRD population. Cohort entry corresponded to the day of the first diagnosis of chronic AF or atrial flutter. Patients were required to have at least one year of medical history in the GPRD prior to cohort entry. We excluded patients with a history of mitral/aortic valve repair/replacement, or hyperthyroidism (either a diagnosis or treatment) at any time prior to cohort entry. All patients were followed until the first of the following events: ischemic stroke or ICH (depending on the outcome being studied), death, end of registration with the general practice, or end of the study period (December 31, 2008).

### Analytic approach

Two nested case–control analyses were performed within the cohort defined above, each corresponding to one of the study outcomes (ischemic stroke and ICH), which were identified on the basis of Read codes. This approach was used because of the time-varying nature of the exposures, the size of the cohort, and the long duration of follow-up [16]. In comparison to a full cohort approach using a survival analysis with time-dependent variables, a nested case–control analysis is computationally more efficient [17], while producing odds ratios (ORs) that are unbiased estimators of incidence rate ratios (RRs) with little or no loss in precision [16-18].

We identified all cases occurring during follow-up, and the calendar date of each case’s event was defined as the index date. Up to 10 controls were randomly selected from the case's risk set, after matching on year of birth, sex, and date of cohort entry. The date of the risk set was the index date for the controls.

### Exposure assessment

For the purposes of this study, we created an algorithm that simultaneously estimates warfarin exposure and therapeutic range at any point in time during follow-up. This algorithm is an adaptation of two algorithms commonly used in AF studies, one devised by Go et al. [19] (warfarin exposure) and Rosendaal et al. [20] (time in therapeutic range). Briefly, patients were considered exposed to warfarin in the presence of a prescription and/or with an international normalized ratio (INR) measurement performed in the outpatient setting. The latter was also used to bridge gaps between any two periods of warfarin prescription coverage. Simple linear interpolation to classify each day of follow-up in predefined categories of therapeutic range (INR: <2, INR: 2 – 3, INR: >3, and INR unknown). A detailed description of this algorithm can be found in the Additional file 1.

Using the algorithm above, cases and controls were classified as to whether they were *current* users of warfarin at index date, while *current* use of other antithrombotic therapies (such as aspirin and other antiplatelets) was defined by the presence of prescriptions in the 90 days prior to index date. Thus, cases and controls were classified into one of five mutually exclusive exposure groups: 1) warfarin monotherapy, 2) aspirin monotherapy, 3) other antithrombotic therapies (such as clopidogrel) or combinations, 4) past use of any antithrombotic therapy (not *current*, but evidence of use in the year prior to index date), and 5) no use of any antithrombotic therapy in the year prior to index date. Current warfarin monotherapy users were further classified according to their anticoagulation level at index date.

### Net clinical benefit

Warfarin and aspirin have been shown to decrease the risk of ischemic strokes (benefits), while increasing the risk of hemorrhage (risks), with ICH being one of the most fatal events, in patients with AF. The relationship between the benefits and risks of these therapies can be expressed according to the following mathematical formula:

Net Clinical Benefit = Adjusted rate difference for stroke _off therapy-on therapy_ – Weight x Adjusted rate difference for ICH _on therapy-off therapy_

For both types of outcomes, we calculated adjusted rate differences (RD) and corresponding 95% confidence intervals using the following formula: R_0_*(1 - RR) for ischemic stroke, and R_0_*(RR - 1) for ICH, where R_0_ was the overall rate of each outcome in the cohort, and RR was the adjusted rate ratio for the relevant outcome in relation to exposure. As with the study by Singer et al [8], the adjusted RDs for ICH were multiplied by a weighting factor, reflecting its relative impact on morbidity and mortality. For comparability with the previous study [8], we used a factor of 1.5 for ICH, and performed sensitivity analyses with weights of 1.0 and 2.0.

### Statistical analysis

Conditional logistic regression was used to estimate RRs along with 95% confidence intervals for ischemic stroke and ICH associated with current use of warfarin monotherapy, overall and according to therapeutic range, and aspirin monotherapy. The reference category for all analyses consisted of no exposure to any antithrombotic therapy in the year prior to index date. In addition to age, sex, year of cohort entry, on which the logistic regression was conditioned, all models were adjusted for the potential confounders listed in Table 1. The stroke model was additionally adjusted for CHADS_2_ score [21], while the ICH model was additionally adjusted for the components of that score at index date.

**Table 1:** Characteristics of cases and matched controls at index date

	**Ischemic stroke**		**Intracranial hemorrhage**	
	**Cases (n = 5519)**	**Controls (n = 55,022)**	**Cases (n = 689)**	**Controls (n = 6858)**
Age, years, mean (SD)*	79.5 (9.2)	79.5 (9.1)	78.2 (9.6)	78.2 (9.4)
Males, n (%)*	2503 (45.4)	24,979 (45.4)	366 (53.1)	3649 (53.2)
Excessive alcohol use, n (%)	76 (1.4)	643 (1.2)	14 (2.0)	106 (1.5)
Smoking status, n (%)				
Ever	2253 (40.8)	22,044 (40.1)	357 (51.8)	3240 (47.2)
Never	2709 (49.1)	28,181 (51.2)	291 (42.2)	3237 (47.2)
Unknown	557 (10.1)	4797 (8.7)	41 (6.0)	381 (5.6)
Obesity, n (%)				
BMI < 30	3407 (61.7)	34,630 (62.9)	445 (64.6)	4575 (66.7)
BMI ≥ 30	810 (14.7)	8716 (15.8)	116 (16.8)	1225 (17.9)
Unknown	1302 (23.6)	11,676 (21.2)	128 (18.6)	1058 (15.4)
Congestive heart failure, n (%)	1432 (25.9)	13,976 (25.4)	163 (23.7)	1625 (23.7)
Hypertension, n (%)	2992 (54.2)	28,238 (51.3)	414 (60.1)	3676 (53.6)
Diabetes, (%)	528 (9.6)	4458 (8.1)	77 (11.2)	640 (9.3)
Prior strokes, n (%)	894 (16.2)	3266 (5.9)	75 (10.9)	408 (5.9)
Peripheral artery disease, n (%)	297 (5.4)	2275 (4.1)	33 (4.8)	293 (4.3)
Myocardial infarction, n (%)	696 (12.6)	6554 (11.9)	83 (12.0)	845 (12.3)
Previous cancer, n (%)	1003 (18.2)	10,605 (19.3)	155 (22.5)	1351 (19.7)
Prior bleeds, n (%)	996 (18.0)	9766 (17.7)	164 (23.8)	1237 (18.0)
Venous thromboembolism, n (%)	421 (7.6)	4094 (7.4)	60 (8.7)	525 (7.7)
ACE inhibitors, n (%)	1738 (31.5)	18,237 (33.1)	259 (37.6)	2565 (37.4)
Angiotensin receptor blockers, n (%)	397 (7.2)	4572 (8.3)	63 (9.1)	756 (11.0)
Antidepressants, n (%)	706 (12.8)	5404 (9.8)	100 (14.5)	725 (10.6)
Antipsychotics, n (%)	555 (10.1)	3858 (7.0)	52 (7.5)	429 (6.3)
NSAIDs, n (%)	981 (17.8)	9102 (16.5)	88 (12.8)	1001 (14.6)
Statins, n (%)	1294 (23.4)	12,503 (22.7)	211 (30.6)	2032 (29.6)

To assess the net clinical benefit of warfarin and aspirin monotherapy, we calculated adjusted RDs which were then used to compute the annualized net clinical benefit of warfarin and aspirin, along with 95% confidence intervals. We also conducted three subgroup analyses to determine whether the net clinical benefit of warfarin and aspirin varied across different groups of patients. For these analyses, cases and matched controls were stratified according to whether they had a history of ischemic stroke, history of any bleed, and CHADS_2_ score at baseline. Finally, in a fourth analysis, we assessed whether the net clinical benefit of warfarin varied as a function of its duration of use. For this analysis, current warfarin monotherapy users were categorized into five groups of continuous use (<3 months, 3–6 months, 6–9 months, 9–12 months, ≥12 months). All analyses were conducted using SAS version 9.2 (SAS Institute Inc., Cary, NC).

## Results

**Figure 1:**
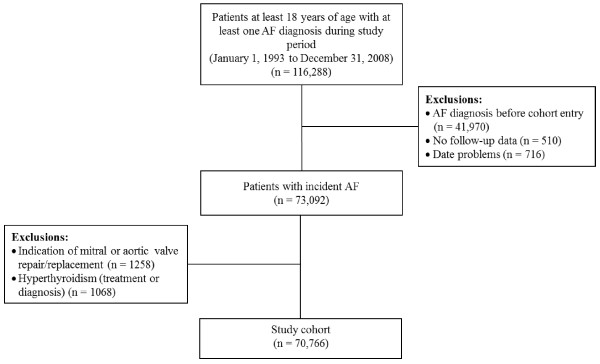
Study flow chart.

A total of 70,766 patients met the inclusion criteria (Figure 1). The mean age at cohort entry was 74.1 (SD: 11.8) years, 51.8% were males, and the mean duration of follow-up was 3.9 (SD: 3.3) years. There were a total of 275,987 person-years of follow-up, during which time 5519 patients experienced an ischemic stroke (overall rate: 2.00% (95% CI: 1.95%, 2.05%) per year), and 689 patients experienced an ICH (overall rate: 0.25% (95% CI: 0.23%, 0.27%) per year). A total of 35,216 (49.8%) patients were prescribed warfarin at least once during follow-up. In terms of CHADS_2_ score at baseline, 21.6% had a score of 0, 33.9% had a score of 1, 26.0% had a score of 2, 10.5% had a score of 3, and 7.9% had a score of ≥ 4. Table 1 presents the characteristics of the cases and matched controls.

As shown in Table 2, current warfarin use was associated with a 35% decreased risk of ischemic stroke compared to no use of any antithrombotic therapy. This effect was driven by being within therapeutic range (adjusted RR: 0.69, 95% CI: 0.57, 0.83), and having an unknown therapeutic range (adjusted RR: 0.62, 95% CI: 0.56, 0.69). Current use of aspirin was not associated with a decreased risk of ischemic stroke. With respect to ICH, an increased risk was only observed in warfarin users above therapeutic range (adjusted RR: 3.26, 95% CI: 1.67, 6.38).

**Table 2:** Adjusted rate ratios of cerebrovascular outcomes associated with the use of warfarin and aspirin

	**Cases/Controls**	**Crude RR**	Adjusted RR (95 % CI)*
**Ischemic stroke**	**5519/55,022**		
No use of any therapy	1513/15,499	1.00	1.00 (reference)
Current use of warfarin monotherapy	896/13,238	0.67	0.65 (0.59, 0.71)
Below therapeutic range (INR: <2)^†^	63/667	0.95	0.93 (0.71, 1.22)
Within therapeutic range (INR: 2–3)^†^	132/1838	0.71	0.69 (0.57, 0.83)
Above therapeutic range (INR: >3)^†^	31/361	0.86	0.82 (0.57, 1.20)
Unknown therapeutic range^†^	670/10,372	0.64	0.62 (0.56, 0.69)
Current use of aspirin monotherapy	2002/18,399	1.11	1.05 (0.98, 1.13)
**Intracranial hemorrhage**	**689/6858**		
No use of any therapy	114/1365	1.00	1.00 (reference)
Current use of warfarin monotherapy	242/2214	1.41	1.29 (1.00, 1.68)
Below therapeutic range (INR: <2)^†^	13/126	1.32	1.16 (0.62, 2.16)
Within therapeutic range (INR: 2–3)^†^	34/356	1.25	1.13 (0.74, 1.72)
Above therapeutic range (INR: >3)^†^	13/47	3.63	3.26 (1.67, 6.38)
Unknown therapeutic range^†^	182/1685	1.39	1.29 (0.98, 1.69)
Current use of aspirin monotherapy	172/2210	0.97	0.92 (0.70, 1.19)

Abbreviations: RR: Rate ratio; CI: Confidence interval; INR: International normalized ratio.

*Adjusted for excessive alcohol use, smoking status, obesity, peripheral artery disease, myocardial infarction, previous cancer, prior bleeds, thromboembolic disorders, ACE inhibitor use, angiotensin receptor blocker use, antidepressant use, antipsychotic use, NSAID use, and statin use. The stroke model was additionally adjusted for CHADS_2_ score, while the intracranial hemorrhage model was additionally adjusted for the components of that score.

^†^Mutually exclusive categories among current users of warfarin monotherapy.

Note: Current users of other antithrombotic therapies or combinations, as well as past users are not displayed in the Table, but were included in the regression model to allow the proper estimation of treatment effects. These represent 1108 cases and 7886 controls for the ischemic stroke model, and 161 cases and 1069 controls for the intracranial hemorrhage model.

### Net clinical benefit of warfarin and aspirin

Table 3 presents the net clinical benefit of warfarin and aspirin, expressed in number of ischemic strokes prevented per 100 persons per year. Based on a weight of 1.5, warfarin monotherapy was associated with a net clinical benefit of 0.59 (95% CI: 0.45, 0.73). This net clinical benefit was mainly driven by patients within range, and those with an unknown therapeutic range (Table 3). Overall, aspirin was not associated with a statistically significant net clinical benefit. Sensitivity analyses using ICH weights of 1.0 and 2.0 produced similar results.

**Table 3:** Net clinical benefit of warfarin, stratified according to anticoagulation intensity, and aspirin

	Adjusted RD*†		**Net clinical benefit (strokes prevented per 100 persons/year) (95 % CI)**		
	**Ischemic stroke ǂ**	**Intracranial hemorrhage ǂ**	**Weight = 1**	**Weight = 1.5**	**Weight = 2**
Current use of warfarin monotherapy	0.70 (0.58, 0.82)	0.07 (0.00, 0.15)	0.63 (0.49, 0.77)	0.59 (0.45, 0.73)	0.56 (0.41, 0.70)
Below therapeutic range (INR: <2)	0.14 (−0.30, 0.58)	0.04 (−0.09, 0.17)	0.10 (−0.36, 0.56)	0.08 (−0.38, 0.54)	0.06 (−0.40, 0.52)
Within therapeutic range (INR: 2–3)	0.62 (0.38, 0.86)	0.03 (−0.06, 0.13)	0.59 (0.33, 0.85)	0.57 (0.31, 0.83)	0.56 (0.30, 0.81)
Above therapeutic range (INR: >3)	0.36 (−0.14, 0.86)	0.56 (0.16, 0.96)	−0.20 (−0.84, 0.44)	−0.49 (−1.13, 0.15)	−0.77 (−1.41, -0.13)
Unknown therapeutic range	0.76 (0.64, 0.88)	0.07 (−0.01, 0.15)	0.69 (0.54, 0.83)	0.65 (0.51, 0.80)	0.62 (0.47, 0.76)
Current use of aspirin monotherapy	−0.10 (−0.24, 0.04)	−0.02 (−0.07, 0.03)	−0.08 (−0.23, 0.07)	−0.07 (−0.22, 0.08)	−0.06 (−0.21, 0.09)

Abbreviations: RD: Rate difference; CI: Confidence interval; INR: International normalized ratio.

* Per 100 persons per year.

† Adjusted for excessive alcohol use, smoking status, obesity, peripheral artery disease, myocardial infarction, previous cancer, prior bleeds, thromboembolic disorders, ACE inhibitor use, angiotensin receptor blocker use, antidepressant use, antipsychotic use, NSAID use, and statin use. The stroke model was additionally adjusted for CHADS_2_ score, while the intracranial hemorrhage model was additionally adjusted for the components of that score.

\**ǂ** RDs for ischemic stroke calculated as the rate when *off* versus *on* therapy, while the RDs for intracranial hemorrhage calculated as the rate when *on* versus *off* therapy.

In subgroup analyses, a high net clinical benefit of warfarin was observed in patients with a history of ischemic stroke (3.34, 95% CI: 2.01, 4.67), although a more modest benefit was also observed in patients with no such history (0.52, 95% CI: 0.38, 0.66). A similar pattern was observed for aspirin, where a net clinical benefit was observed in patients with a history of ischemic stroke (1.69, 95% CI: 0.04, 3.33), while no benefit was observed in those with no such history (−0.07, 95% CI: -0.22, 0.08). In contrast, patients with no history of bleeds were the ones most likely to benefit from warfarin therapy (0.65, 95% CI: 0.50, 0.80), while patients with a history of bleeds had no benefit (0.40, 95% CI: -0.11, 0.91). With respect to aspirin, no net clinical benefit was observed in either those without and with a history of bleeds (−0.05, 95% CI: -0.21, 0.11 and −0.08, 95% CI: -0.61, 0.46, respectively).

**Figure 2:**
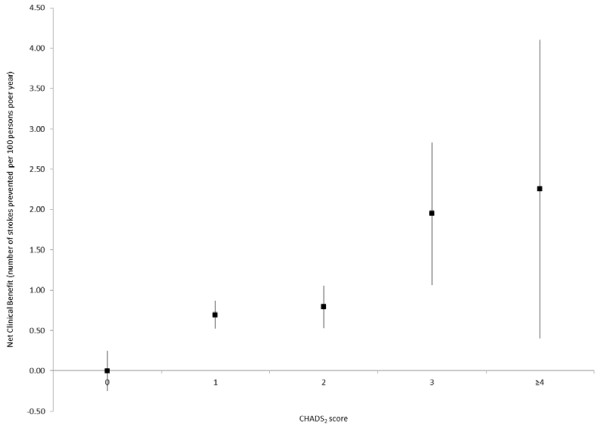
** Net clinical benefit of warfarin, stratified according to CHADS**_**2**_**score at baseline (based on an ICH weight of 1.5).**

**Figure 3:**
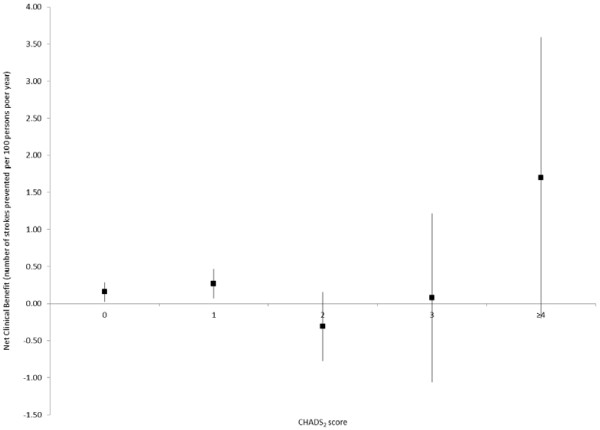
** Net clinical benefit of aspirin, stratified according to CHADS**_**2**_**score at baseline (based on an ICH weight of 1.5).**

**Figure 4:**
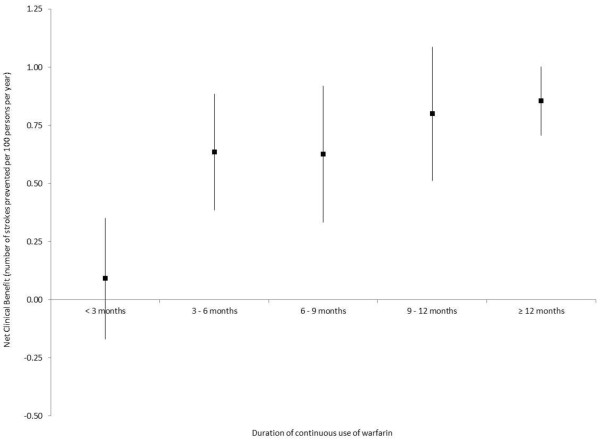
Net clinical benefit of warfarin according to duration of continuous use (based on an ICH weight of 1.5).

As shown in Figure 2, the net clinical benefit of warfarin increased as the CHADS_2_ score increased. With respect to aspirin, a modest net clinical benefit was observed in patients with CHADS_2_ scores of 0 and 1 at baseline, while no statistically significant net clinical benefit was observed with other CHADS_2_ scores (Figure 3). Finally, the net clinical benefit of warfarin became apparent after 3 months of continuous use and was maintained with longer duration of use (Figure 4).

## Discussion

The results of this large population-based study indicate that warfarin is associated with a net clinical benefit in preventing ischemic strokes in patients with AF, while overall, no net clinical benefit is observed with aspirin. Furthermore, this study presents for the first time, the net clinical benefit of warfarin classified according to different levels of anticoagulation. Our results confirm that the net clinical benefit of warfarin is limited to patients within therapeutic range, and thus emphasize the need to adequately maintain patients within that range. While our results confirm previous findings [8,9] that high risk patients, such as those with a history of ischemic stroke and high CHADS_2_ scores are those most likely to benefit from this therapy, they also justify physician concerns that warfarin may not be indicated for patients with a history of bleeds. Similar patterns were observed with aspirin in subgroup analyses, although the net clinical benefits were more modest than those observed with warfarin. Finally, our results indicate that the net clinical benefit of warfarin becomes apparent only after 3 months of continuous use, highlighting the importance of improving treatment persistence in patients using this therapy.

To our knowledge, this is one of the largest population-based studies to have evaluated the net clinical benefit of warfarin and aspirin in patients with AF. We observed that only 50% of the cohort was ever exposed to warfarin during follow-up, demonstrating underutilization which is consistent with other studies [4-6,22,23]. We also found that patients with a history of stroke and those without a history of bleeds were the ones most likely to benefit from warfarin therapy, thus identifying populations for whom this therapy may or may not be appropriate. Finally, the net clinical benefit of warfarin was maintained with longer periods of continuous use. This novel finding emphasizes the need to improve treatment persistence, as 26% to 30% of patients with AF discontinue warfarin within the first year of treatment [24,25].

As observed by others [9], we did not find any net clinical benefit with aspirin overall. However, we did observe a net clinical benefit in patients with low CHADS_2_ scores (0 and 1), populations for whom aspirin is typically indicated. These net clinical benefits were however, lower than those observed with warfarin. Finally, while aspirin had a net clinical benefit in patients with a history of stroke, no benefit was observed in those with no history of bleeds. Overall, our aspirin results need to be interpreted with caution. Unlike warfarin, aspirin is available over-the-counter, possibly leading to exposure misclassifications. While chronic aspirin users typically receive prescriptions for this drug, which is dispensed almost free of charge in in the UK population, it is possible that some patients were misclassified as unexposed, leading to an underestimation of an already weak treatment effect.

This population-based study has a number of strengths and some potential limitations. This was a large population-based cohort of patients with AF, followed for up to 16 years, enabling the identification of a large number of cases. In addition, because medical and drug information in the GPRD is prospectively collected, the possibility of recall bias was eliminated. However, drug information in the GPRD represents prescriptions written by general practitioners. As such, it is unknown whether prescriptions were actually filled at the pharmacy and whether patients fully adhered with the treatment regimen. Such non-differential misclassification of exposure would have biased the results towards the null. Another limitation is that stroke events may be underreported in the GPRD, which would lead to an underestimation of the treatment effects. Furthermore, ischemic strokes were defined on the basis of a specific diagnostic code for this event or a diagnostic code of ‘stroke’ with no mention of the subtype. Since stroke subtypes are not always specified in the GPRD files, it is possible that some hemorrhagic strokes were misclassified as being ischemic. However, this potential bias is likely to have been minimal as the vast majority of strokes are ischemic (>80%), and our overall rate was very similar to the one reported in the previous study (2.0% per year versus 2.1% per year, respectively) [8]. Finally, as with any observational study, confounding by indication is a concern, whereby the risk profile of patients prescribed warfarin or aspirin is likely to be different from the one of those not prescribed any antithrombotic therapy. Although we adjusted for a number of potential confounding factors including BMI, excessive alcohol use, and smoking which are often absent in administrative databases, residual confounding may still be present.

We assessed the net clinical benefit of warfarin and aspirin on the basis of comparing their benefits in ischemic stroke prevention to their risks in increasing the incidence of ICH. However, it would have been of interest to compare other potential benefits (such as prevention of MI in warfarin users [26]) to other risks associated with these therapies, such as major bleeding events (other than ICH). Unfortunately, the GPRD does not collect detailed information to objectively classify the severity of bleeding events (such as those events requiring hospitalization, and those associated with decreases in hemoglobin, or those requiring red blood cell transfusions). Additional studies are needed to consider the net clinical benefit of warfarin and aspirin in the context of a broader range of outcomes.

In summary, our study provides the net clinical benefit of warfarin and aspirin in patients with AF in the natural setting of clinical practice. Our results indicate that patients taking warfarin outside of the recommended therapeutic range are unlikely to benefit from this therapy, while aspirin confers a weak net clinical benefit in selected populations. Finally, our findings emphasize the need to identify high risk populations that would benefit the most from warfarin therapy, and ensure that such patients are maintained within therapeutic range, while identifying strategies to improve treatment persistence.

## Abbreviations

AF, Atrial fibrillation; CI, Confidence interval; GPRD, General Practice Research Database; ICH, Intracranial hemorrhage; INR, International normalized ratio; RCT, Randomized controlled trial; RD, Rate difference; RR, Rate ratio; UK, United Kingdom.

## Competing interests

LA, SD, and CR have no conflicts of interest to declare. TS is an employee of Bristol-Myers Squibb. SS has received funding and has participated in advisory committees for Bristol-Myers Squibb, Boehringer-Ingelheim and Bayer-Schering.

## Authors’ contributions

LA, SD, and SS constructed the concept and design of the project, performed the analysis, interpreted the data, drafted the manuscript and approved the final manuscript to be published. All authors participated in the analysis and interpretation of data, revised the manuscript critically for important intellectual content and approved the final manuscript to be published.

## Pre-publication history

The pre-publication history for this paper can be accessed here:

http://www.biomedcentral.com/1471-2261/12/49/prepub

## Supplementary Material

Additional file 1 Algorithm to estimate warfarin exposure and therapeutic range. Click here for file
