# Supplementary material for: A net clinical benefit analysis of warfarin and aspirin on stroke in patients with atrial fibrillation: a nested case–control study
Source: BMC Cardiovasc Disord. 2012 Jun 26;12:49. doi: 10.1186/1471-2261-12-49 (PMC3444325; doi:10.1186/1471-2261-12-49)
Supplement: Additional file 1 — Algorithm to estimate warfarin exposure and therapeutic range. [file 1471-2261-12-49-S1.doc]

**Additional Material file**

**Algorithm to estimate warfarin exposure and therapeutic range**

**Warfarin exposure**

An algorithm was created to identify periods of *warfarin exposure* for all cohort members. In the simplest scenario, warfarin exposure began with a first warfarin prescription. The duration of a *prescription coverage* corresponded to the date of the prescription plus its specified duration of use. Patients were considered to be continuously exposed to warfarin if a new prescription overlapped with the prescription coverage of a previous one. In addition, we considered a 30-day grace period following the end of consecutive prescription coverage, to allow for modifications in weekly dose that may not have been reflected in the original prescription, plus a 15-day elimination period, corresponding to the five half-life elimination period of warfarin from the body. Thus, a *warfarin exposure period* corresponded to the total prescription coverage plus a 30-day grace period and a 15-day elimination period (Figure 1a).

Exposure to warfarin in the scenario above is based on the presence of warfarin prescriptions. However, there are certain situations in which patients may have been exposed to warfarin, but their prescriptions were not recorded in the database. Such exposed patients would be classified as unexposed, thereby introducing exposure misclassification. To minimize misclassification bias, INRs served two important roles in this new algorithm.

The first was to account for the possibility that hospitalized patients may have received a discharge prescription for warfarin. Since the database does not capture drugs prescribed during hospitalizations, such patients would have been misclassified as unexposed up until the appearance of a warfarin prescription in the database. To minimize this bias, warfarin exposure coverage could have begun with an INR, as an indicator that warfarin was prescribed and patients were being monitored. Therefore, patients were assumed to be on warfarin up to 45 days (based on the 30-day grace period and 15-day elimination period) after the date of that first INR (Figure 1b).

In its second role, INRs were used to bridge gaps between any two periods of warfarin prescription coverage. Such gaps could occur if patients undergoing warfarin therapy were followed outside their general practice, such as in anticoagulation clinics. For such patients, prescription information would have been incompletely captured in the database. While this may have occurred, it was possible for GPs to receive INR information from the anticoagulation clinics. When gaps occurred, we searched for the last INR occurring prior to the end of a warfarin exposure period, and extended that exposure period an additional 45 days from the date of that last INR. Multiple INR measurements less than or equal to 45 days apart were bridged and defined a more extended period of warfarin exposure (Figure 1c).

**Time in therapeutic range**

Once a *warfarin exposure period* was determined as described above, it was of interest to classify exposure periods in terms of person-time spent in therapeutic range. Therefore, a second aspect of the algorithm was created, to classify the person-time spent in different categories of therapeutic range using reported INR results.

*Start of warfarin exposure period*

The first step was to determine whether there was an INR measurement at the start of a warfarin exposure period. When that was the case, that INR measurement was used to calculate time in therapeutic range by the method described below. If there was no INR measurement available at the start of a warfarin exposure period, but INRs were present ≤ 45 days after the start of a warfarin exposure period, an INR of 1.0 was set at the beginning of the warfarin exposure period (Figure 2, Exposure Period a). If there were no INRs within the 45 days after the start of a warfarin exposure period, that time interval was classified as “unknown” up until the presence of an INR measurement occurring during the warfarin exposure period (Figure 2, Exposure Period b).

*Simple linear interpolation*

Simple linear interpolation was used to determine the person-time spent in predefined categories of therapeutic range (INR: <2, INR: 2 – 3, INR: >3, and INR unknown) between two INR measurements. Simple linear interpolation was used only if there was ≤ 45 days separating two consecutive INR measurements. If the time interval between two consecutive INR measurements was longer than 45 days, person-time was classified according to the following:

If consecutive INR measurements during a warfarin exposure period were separated by more than 45 days, the value of the first INR was carried forward 45 days. Starting at day 46, person-time was classified as “unknown”, up until the appearance of the next INR measurement occurring during that warfarin exposure period (Figure 2, Exposure Period b). The same (carry forward for 45 days) approach was used for the last INR measurement during a warfarin exposure period if it occurred more than 45 days before the end of the warfarin exposure period (Figure 2, Exposure Period c).

*End of warfarin exposure period*

If the last INR during a warfarin exposure period occurred during a period of *prescription warfarin exposure*, then an extrapolated INR value was set to 1.0 and assigned to the date 45 days after the last recorded INR. If the last INR during a warfarin exposure period was a “bridging” INR – that is, one that did not occur at a time when there was *prescription warfarin exposure*, then the value of that last INR was carried forward 45 days (Figure 2, Exposure Periods a, b, and c).

**Figure 1**

**Figure 2**
